# Reproducible preclinical research—Is embracing variability the answer?

**DOI:** 10.1371/journal.pbio.2005413

**Published:** 2018-03-05

**Authors:** Natasha A. Karp

**Affiliations:** Quantitative Biology, Discovery Sciences, IMED Biotech Unit, AstraZeneca, Cambridge, United Kingdom

## Abstract

Translational failures and replication issues of published research are undermining preclinical research and, if the outcomes are questionable, raise ethical implications over the continued use of animals. Standardization of procedures, environmental conditions, and genetic background has traditionally been proposed as the gold standard approach, as it reduces variability, thereby enhancing sensitivity and supporting reproducibility when the environment is defined precisely. An alternative view is that standardization can identify idiosyncratic effects and hence decrease reproducibility. In support of this alternative view, Voelkl and colleagues present evidence from resampling a large quantity of research data exploring a variety of treatments. They demonstrate that by implementing multi-laboratory experiments with as few as two sites, we can increase reproducibility by embracing variation without increasing the sample size.

## A reproducibility crisis

Across all fields of scientific research, scientists are concerned by the inability to replicate published research, and this is increasingly seen as a problem with the scientific method [1]. There is an active debate on what reproducibility means [2]; within this article, we are focusing on the ability to repeat a study independently with a similar setup and make a similar conclusion (called replicability or results reproducibility). The reproducibility problem is multifaceted and includes issues from the initial collection of data (experimental design, low power, poorly validated outcome variables or models), to data analysis, to a lack of replication, and in reporting (selective reporting, insufficient details) [3]. The importance of these issues has led to a refinement of the definition of the reduction element of the Three Rs (3Rs) guiding framework for the ethical use of animals by the National Centre for the Replacement Refinement and Reduction of Animals in Research (NC3Rs). The reduction element, which has historically focused on minimizing the number of animals used per experiment, has been refined to “appropriately designed and analyzed animal experiments that are robust and reproducible, and truly add to the knowledge base” [4].

When testing a hypothesis by running experiments with control and treated groups, the scientific method is fundamentally trying to determine causality. Historically, we have focused our efforts on ensuring high internal validity, such that we have high confidence that any change detected was due to the treatment and no other explanation could explain the observations. Experiments that are highly controlled using standardization for known sources of variation and utilizing randomization and blinding to remove potential confounders are considered the gold standard of research. Standardization was promoted as a means of reducing variation within an experiment, thereby increasing sensitivity. External validity, in contrast, considers the extent to which the results of a study can be generalized to other situations and is harder to achieve. To address reproducibility issues, attention has traditionally focused on using standardization and reporting in detail the methodologies used.

## The challenge of phenotypic plasticity

**Fig 1:**
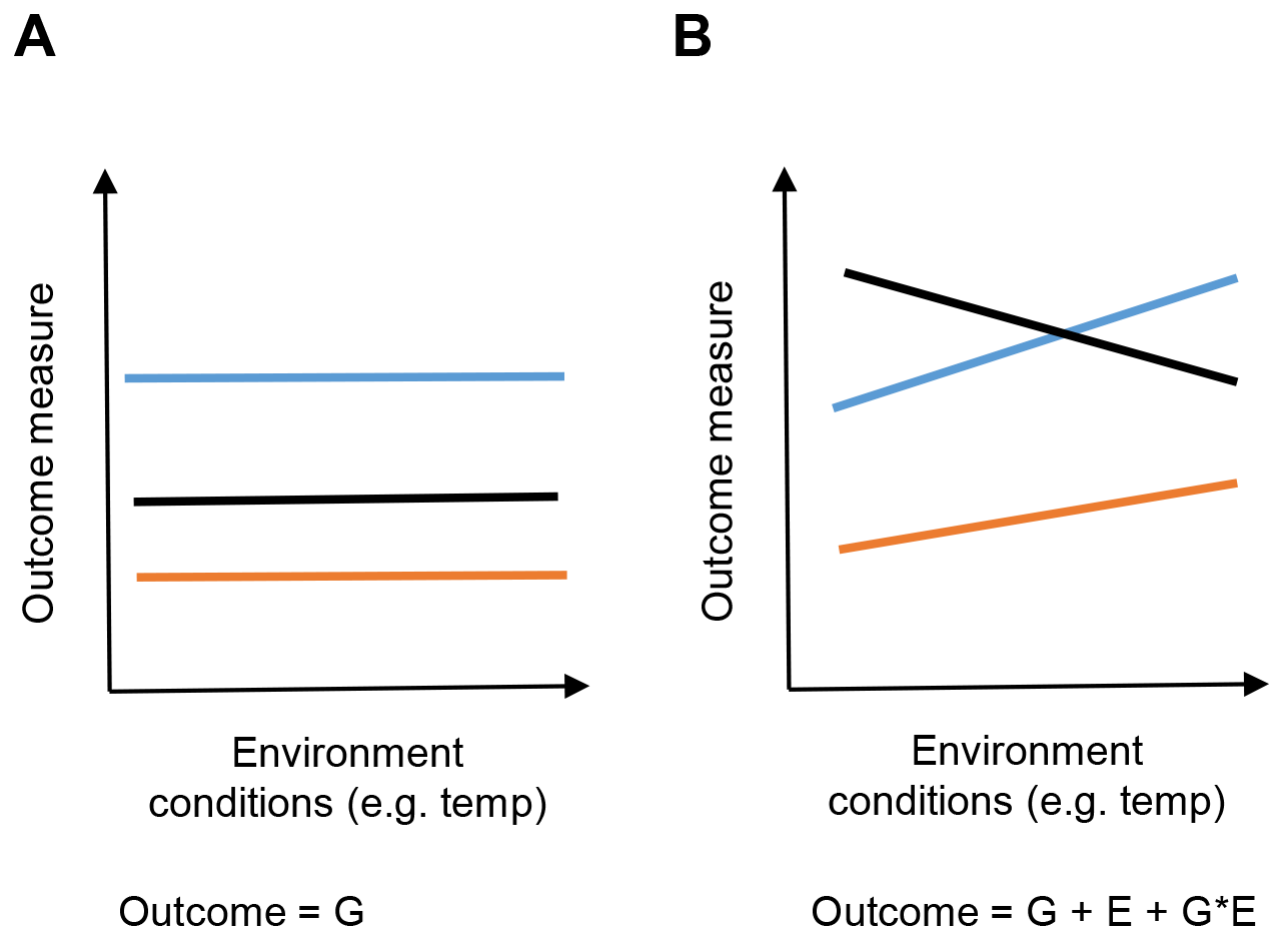
Visualization of phenotypic plasticity. A schematic to demonstrate the effect of phenotypic plasticity on an outcome measure. Each line with a unique color represents a different genotype. (A) Behavior when there is no phenotypic plasticity showing an outcome that depends on the genotype but is independent of the environment. (B) Behavior when there is phenotypic plasticity showing that the outcome depends on the genotype, environment, and an interaction between the genotype and environment. The observed phenotype for an organism, for the majority of traits, is a function of the environment and the genotype. G, genotype; E, environment; temp, temperature.

Preclinical research has an additional challenge in that living organisms are highly responsive to the environment; this ability has been described as phenotypic plasticity. The capacity to change in response to the environment is fundamental to an organism’s survival ability and is thought to be an evolutionary adaptation, allowing individuals to “fit” their phenotype to different environments (Fig 1) [5]. In a groundbreaking study, scientists went to great lengths to standardize the environment and protocols in a characterization of multiple mice strains within three laboratories with a number of behavioral screens. Despite this extensive standardization, they observed disparate results and proposed that interactions between the genotype and local environment lead to idiosyncratic phenotypes [6]. These findings led to a further call to standardize [7]. However, recent research has shown that, even in highly standardized environments, phenotypes in control mice fluctuate unexpectedly between batches [8]. This indicates that mice are reacting to environmental variation beyond the established known criteria; thus, reproducing the original environment and hence the phenotype can be challenging. This finding was significant because batch-to-batch variation was observed across all traits studied and included physiological assays such as clinical chemistry, and it was not restricted to studying behavioral screens, on which attention has traditionally focused.

**Fig 2:**
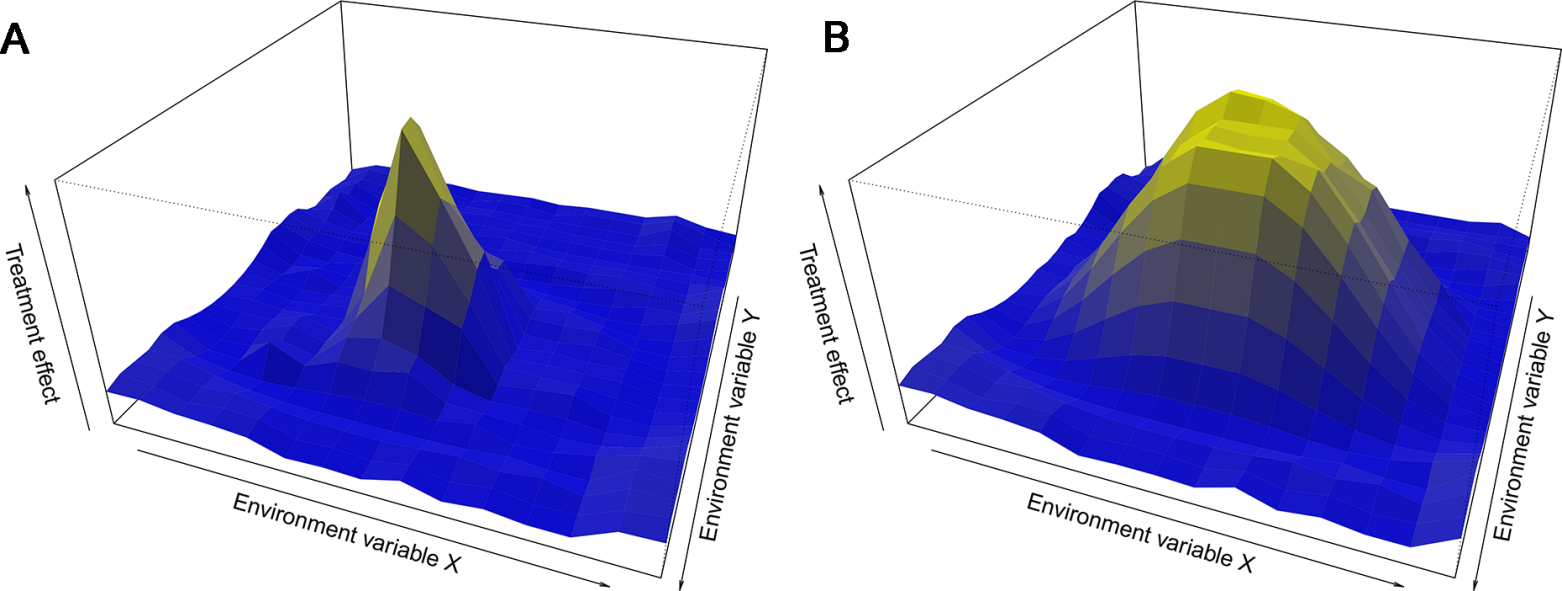
Treatment effect can depend on the environment. The response of an organism to a treatment not only depends on the treatment but also on the state of the organism, which is a function of the current and historic environment. (A) Visualizes a treatment effect that is idiosyncratic to a particular environment. (B) Visualizes a treatment effect that is more generalizable. Whilst idiosyncratic findings are biologically relevant and can give insight to biological function and mechanism, a treatment effect that is more generalizable may be more likely to translate.

Instead of minimizing variation, embracing variability has been argued to make a study more representative and thereby improve external validity (Fig 2). Suggestions have included splitting experiments into multiple independent batches, introducing systematic heterogenization, or implementing multicenter studies. A proof of concept study by Richter and colleagues found that heterogenization on age and housing improved reproducibility of behavioral studies comparing inbred strains of mice [9].

An alternative to systematic heterogenization has been proposed by Kafkafi and colleagues, who used historic data comparing strains from multiple laboratories to estimate a trait’s susceptibility to idiosyncratic findings, and this heterogeneity in response can be used to adjust the significance score for a study [10]. This approach, however, depends on accurately estimating the trait’s susceptibility and, for most traits, is currently unavailable.

## Exploring the impact of multi-laboratory design

In their study reported in this issue of *PLOS Biology*, Voelkl and colleagues, through simulations utilizing real data, assessed the value of embracing variation in the experimental design by introducing variability (multi-laboratory versus single-laboratory studies) while using the same number of animals in total [11]. The findings are generalizable as they utilized a large database of preclinical research, looking across 13 interventions in animal models of stroke, myocardial infarction, and breast cancer. These studies were originally run completely independently and hence included both genetic and environmental variability, as different strains, ages, housing conditions, etc., were used. In their simulations mimicking different experimental designs, they calculated the coverage probability as a measure of reproducibility; an experiment is reproduced if the “true” treatment effect (estimated from meta-analysis of all studies) is included within the 95% confidence interval estimated for that individual experiment. They found that reproducibility substantially increased as the number of laboratories increased, and the biggest improvement occurred when moving from 1 to 2 sites. Furthermore, they found this was further complemented by an increase in sensitivity with a multisite design when experiments were sufficiently powered. The improvement arose from increased accuracy and reduction in variability of effect size estimates, as seen in that there were fewer outlier experiments. There was a small penalty in that the estimate had a large confidence interval, but it could be argued that this higher uncertainty reflects the true uncertainty when the treatment effect is considered in a larger context that encompasses biological variation.

This work is pivotal in the debate on addressing replicability issues in preclinical research, as it demonstrates that systematic heterogenization improves reproducibility and was necessary in nonbehavioral screens. The finding that significant improvement in reproducibility by multi-laboratory design did not require a large number of participating laboratories nor an increase in sample size addresses many of the practical blockers to implementing such a design. This is why the authors recommended that a multi-laboratory approach should be the gold standard for late phase confirmatory preclinical trials.

## Remaining questions and wider implications

This study provides an example of systematic heterogenization increasing reproducibility. The heterogeneity introduced was high and this raises the question of how much heterogeneity would be sufficient to improve the reproducibility. If multisite studies are based on protocols that are harmonized, the benefits of improving reproducibility may be lower than those observed within this publication. Furthermore, ethically and for the efficiency of science, we need to increase reproducibility throughout the research pipeline. This raises the question of how we can practically introduce variation within a single laboratory. Would multiple batches be sufficient? In reality, the introduction of variation will be on spectrum, and the more variation we introduce, the more reliable and translatable the finding will be.

Including variation in a non-systematic way would inflate the number of animals needed and potentially confound the experiment. Instead, an experimental design needs to be developed along with an appropriate analysis pipeline that assesses the treatment effect after accounting for the introduced variation. Examples include split plot designs or randomized block designs and regression analysis with fixed or random effects for the sources of variation included in the design. Unfortunately, it is well recognized that lack of training in both design and analysis hinders biologists [12], and this will impede progress.

To date, ethical review bodies have focused on minimizing harm and typically assume that the validity and reproducibility of the studies are met [13]. This research demonstrates that we need a mind-set change to meet our ethical obligations and consider how we maximize experimental validity, and this presents a new challenge for conducting harm–benefit analysis [14]. Traditionally, the 3Rs framework has emphasized using as few animals as possible; however, we are trading high sensitivity in a narrow window of testing space against generalizability and therefore reducing reproducibility. The need to update our thinking is reflected in the working definition proposed by NC3Rs for reduction, which frames the use of animals not within a single experiment but more globally.

Most of the discussion on the reproducibility crisis has focused on improving internal validity. Whilst the external validity issue is more acute for studies involving living organisms, due to the occurrence of phenotypic plasticity, the prevalence of generalizing beyond the original testing scope is a general issue for the scientific community. It is important to improve internal validity; however, this alone is not going to address the reproducibility crisis in science, and the concept of embracing variation needs to be raised universally.

## Abbreviations

NC3Rs: National Centre for the Replacement Refinement and Reduction of Animals in Research
3Rs: Three Rs

## References

[1] Baker M. Is there a reproducibility crisis? A Nature survey lifts the lid on how researchers view the'crisis rocking science and what they think will help. Nature. 2016;533(7604):452–5. doi: 10.1038/533452a 27225100

[2] Goodman SN, Fanelli D, Ioannidis JP. What does research reproducibility mean? Science translational medicine. 2016;8(341):341ps12–ps12. doi: 10.1126/scitranslmed.aaf5027 27252173 10.1126/scitranslmed.aaf5027

[3] Jarvis MF, Williams M. Irreproducibility in preclinical biomedical research: Perceptions, uncertainties, and knowledge gaps. Trends in pharmacological sciences. 2016;37(4):290–302. doi: 10.1016/j.tips.2015.12.001 26776451 10.1016/j.tips.2015.12.001

[4] NC3Rs. The 3Rs. Available from: www.nc3rs.org.uk/the-3rs. Cited 2018 16th January 2018.

[5] Garland T, Kelly SA. Phenotypic plasticity and experimental evolution. Journal of Experimental Biology. 2006;209(12):2344–61. 16731811 10.1242/jeb.02244

[6] Crabbe JC, Wahlsten D, Dudek BC. Genetics of mouse behavior: interactions with laboratory environment. Science. 1999;284(5420):1670–2. 10356397 10.1126/science.284.5420.1670

[7] Brown SD, Hancock JM, Gates H. Understanding mammalian genetic systems: the challenge of phenotyping in the mouse. PLoS Genet. 2006;2(8):e118 doi: 10.1371/journal.pgen.0020118 16933996 10.1371/journal.pgen.0020118 PMC1557775

[8] Karp NA, Speak AO, White JK, Adams DJ, de Angelis MH, Hérault Y, et al Impact of temporal variation on design and analysis of mouse knockout phenotyping studies. PLoS ONE. 2014;9(10):e111239 doi: 10.1371/journal.pone.0111239 25343444 10.1371/journal.pone.0111239 PMC4208881

[9] Richter SH, Garner JP, Auer C, Kunert J, Würbel H. Systematic variation improves reproducibility of animal experiments. Nature Methods. 2010;7(3):167–8. doi: 10.1038/nmeth0310-167 20195246 10.1038/nmeth0310-167

[10] Kafkafi N, Golani I, Jaljuli I, Morgan H, Sarig T, Würbel H, et al Addressing reproducibility in single-laboratory phenotyping experiments. Nature Methods. 2017;14(5):462–4. doi: 10.1038/nmeth.4259 28448068 10.1038/nmeth.4259

[11] Voelkl B, Vogt L, Sena ES, Wurbel H. (2018) Reproducibility of pre-clinical animal research improves with heterogeneity of study samples. PLoS Biol. 16(2): e2003693 https://doi.org/10.1371/journal.pbio.2003992 29470495 10.1371/journal.pbio.2003693 PMC5823461

[12] Weissgerber TL, Garovic VD, Milin-Lazovic JS, Winham SJ, Obradovic Z, Trzeciakowski JP, et al Reinventing biostatistics education for basic scientists. PLoS Biol. 2016;14(4):e1002430 doi: 10.1371/journal.pbio.1002430 27058055 10.1371/journal.pbio.1002430 PMC4825954

[13] Vogt L, Reichlin TS, Nathues C, Würbel H. Authorization of Animal Experiments Is Based on Confidence Rather than Evidence of Scientific Rigor. PLoS Biol. 2016;14(12):e2000598 doi: 10.1371/journal.pbio.2000598 27911892 10.1371/journal.pbio.2000598 PMC5135031

[14] Würbel H. More than 3Rs: the importance of scientific validity for harm-benefit analysis of animal research. Nature. 2017;20:1. 10.1038/laban.1220 28328898

